# Farm factors associated with increased free fatty acids in bulk tank milk*

**DOI:** 10.3168/jdsc.2022-0301

**Published:** 2022-12-01

**Authors:** Hannah M. Woodhouse, David F. Kelton

**Affiliations:** Department of Population Medicine, University of Guelph, Guelph, ON, Canada N1G2W1

## Abstract

An elevated amount of free fatty acids (FFA) is a milk quality concern that can contribute to off-flavor, rancidity, reduced foaming ability, and inhibited fermentation that affects cheese coagulation. Free fatty acid concentrations >1.2 mmol/100 g of fat are considered high, although there are various thresholds and units used to quantify levels. The FFA result from milk fat breakdown through spontaneous, bacterial, or induced lipolysis. The amount of bulk tank milk FFA can vary between farms as well as within farms daily, and this mini-review aimed to identify those risk factors at the farm level associated with elevated FFA. A search of the literature identified 5 current sources selected for this review based on relevance. Cows that were milked by automated milking systems (AMS) are suggested to produce milk that is higher in FFA compared with conventional parlors. Factors associated with AMS contributing to spontaneous lipolysis include higher milking frequencies, reduced milking intervals, and low milk yields at each robot visit. Automated milking systems also have characteristics of quarterly milking and high milk lines that can increase vacuum fluctuations and air admission contributing to induced lipolysis. Both AMS and conventional systems with poor tank cooling or without precooling mechanisms can be at risk for higher bulk tank FFA. Bacterial lipolysis can occur when milk temperatures fluctuate and rise, or when there is insufficient milking system cleaning and sanitization. Feed factors such as high saturated fatty acid diets can increase the likelihood of spontaneous lipolysis. We concluded that the major factors associated with increased levels of FFA are non-parlor milking systems, increased air admission, the absence of additional cooling, temperature fluctuations in the bulk tank, and rations high in saturated fatty acids. Future research further investigating these factors can help to minimize FFA and ensure milk quality.

## Highlights

• Free fatty acids (FFA) result from milk fat breakdown.
• The presence of elevated FFA (>1.2 mmol/100 g of fat) is a milk quality concern.
• FFA concentration is multifactorial and many factors arise at the farm level.
• These factors stem from milk production, harvest, transport, and storage.

## Graphical Abstract

**Figure:**
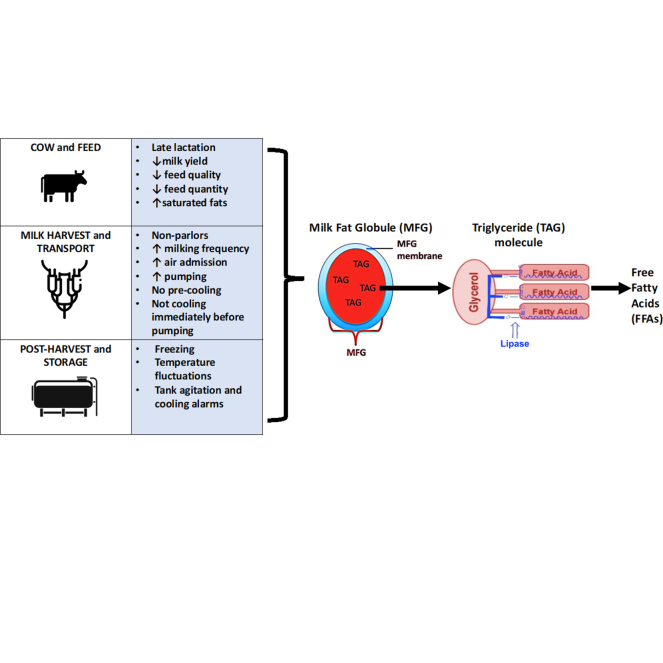
**Summary:** Elevations in free fatty acids (FFA) in milk can result from multiple farm factors. These include cow characteristics, ration composition, milk harvest equipment, milk transport, and milk storage. Although through different mechanisms, each of these factors can result in increased lipase enzyme breakdown of triacylglycerol (TAG) molecules contained inside milk fat globules. The result is a release of FFA that, when in high concentrations, can negatively affect the quality of dairy products.

Milk quality is arguably the most important aspect of milk production. Consumer satisfaction in dairy products is key to the sustainability of the dairy industry. Milk quality has traditionally been defined in terms of the absence of antibiotics and hormones as well as low somatic cell and bacteria counts. However, several European researchers suggest that free fatty acid (**FFA**) concentration should also be considered a milk quality attribute (de Koning et al., 2003; Wiking et al., 2005, 2006, 2019; Rasmussen et al., 2006). This is driven by the fact that elevated FFA concentrations in milk are associated with off-flavor, rancidity, reduced foaming ability, and inhibited milk fermentation that affects cheese coagulation (de Koning et al., 2003; Wiking et al., 2005, 2006, 2019).

Fat is an important component of milk, with most existing in the form of triglycerides (or triacylglycerol, **TAG**) (Wiking et al., 2019). Free fatty acids are products of fat breakdown by lipolysis (de Koning et al., 2003; Wiking et al., 2005, 2006, 2019; Rasmussen et al., 2006), a catabolic process that results in the cleavage of TAG to form 3 fatty acids and a glycerol molecule through hydrolysis (de Koning et al., 2003; Wiking et al., 2006).

There is inconsistency in the literature with respect to a suggested sensory threshold for FFA because fatty acid composition in milk varies, and various analytical techniques are used to quantify their levels (Wiking et al., 2019). Wiking et al. (2019) suggest that any level >1.2 mmol/100 g of fat (as measured by infrared spectroscopy) is concerning and can lead to milk quality issues. de Koning et al. (2003) conducted a study on 124 herds and classified “high FFA” as any concentration >0.78 mmol/100 g of fat. The units for reporting FFA can also vary, but most studies report levels in millimoles per 100 g of fat (mmol/100 g of fat), milliequivalents per 100 g of fat (mEq/100 g of fat), or milliequivalents per liter of milk (mEq/L) (de Koning et al., 2003; Wiking et al., 2005, 2006, 2019; Rasmussen et al., 2006). Although quantifying the sensory threshold of FFA remains a challenge, the consequences on milk quality are well documented.

There are different forms of lipolysis in milk, but the effects of increased FFA are cumulative. Spontaneous lipolysis is catalyzed by lipoprotein lipases (**LPL**) and influenced by the physiological state of the cow and her nutrition (de Koning et al., 2003; Wiking et al., 2005, 2006, 2019; Rasmussen et al., 2006). Bacterial lipolysis is catalyzed by bacterial lipases and is influenced by hygiene during milk harvest and milk cooling (de Koning et al., 2003; Wiking et al., 2019). Induced lipolysis is a result of physical stress on the milk fat globule membrane and is influenced by milk transport and handling factors (de Koning et al., 2003; Wiking et al., 2005; Rasmussen et al., 2006).

Lipoprotein lipase is an endogenous milk enzyme that catalyzes spontaneous lipolysis by cleaving TAG to separate fatty acids from the glycerol molecule (de Koning et al., 2003; Wiking et al., 2005, 2006, 2019). It is produced in the mammary gland and its function is important in the uptake of blood lipids to construct milk fat (Wiking et al., 2006, 2019). Although LPL is abundant in normal milk, a protective milk fat globule membrane prevents its access to the TAG (de Koning et al., 2003; Wiking et al., 2005, 2006, 2019). If the milk fat globule membrane endures mechanical stress or agitation, it can expose hydrophobic sites where casein micelles and whey proteins can adsorb (Wiking et al., 2005). Because LPL is most often associated with the casein micelle, adsorption of casein brings LPL closer to the TAG and risks their cleavage to produce FFA (de Koning et al., 2003; Wiking et al., 2005, 2006, 2019). A similar process occurs in bacterial lipolysis; however, bacterial lipases catalyze the milk fat breakdown instead of LPL (Wiking et al., 2005). For induced lipolysis, physical or chemical damage to the milk fat globule membrane itself exposes the TAG to LPL or bacterial lipases, making them easier to target (de Koning et al., 2003; Wiking et al., 2005; Rasmussen et al., 2006).

The amount of FFA in bulk tank milk leaving the dairy farm varies daily, with this variation resulting from many factors causing spontaneous, bacterial, and induced lipolysis (Wiking et al., 2019). These can be broadly classified as cow factors (genetics, parity, stage of lactation), herd management factors (feed), and milk harvest factors (equipment, milk transfer, storage) (de Koning et al., 2003; Wiking et al., 2005, 2006, 2019; Rasmussen et al., 2006). Although there are likely postharvest elements (transportation and processing) to consider, this review focuses on the on-farm factors affecting the level of FFA in bulk tank milk.

Optimal lactating rations are essential to produce a high yield of high-quality milk that is low in FFA (de Koning et al., 2003; Wiking et al., 2005, 2019; Rasmussen et al., 2006). Underfeeding cows can increase cow stress and the susceptibility of their milk to lipolysis, which could influence FFA (de Koning et al., 2003; Wiking et al., 2019). de Koning et al. (2003) conducted a study on 262 herds from the Netherlands and reported an association between an increased number of water and feeding areas and lower FFA to support this statement.

The composition of the lactating ration also affects FFA concentrations. Feeding a ration higher in saturated fat (either natural or through supplementation) increases milk fat yields, but it can also increase FFA (de Koning et al., 2003; Wiking et al., 2005, 2019; Rasmussen et al., 2006). Saturated fatty acids produce large and unstable TAG that increase the size of the milk fat globule, making it more susceptible to lipolysis (de Koning et al., 2003; Wiking et al., 2005, 2019; Rasmussen et al., 2006).

Lactating ration ingredients can also influence FFA, and it is thought that diets higher in UFA can be protective (de Koning et al., 2003; Wiking et al., 2005, 2019; Rasmussen et al., 2006). In an experimental feeding crossover design that compared lactating rations of 8 cows, lower FFA concentrations were observed in milk of cows on a high-UFA diet compared with a high-SFA diet (Wiking et al., 2005). Higher levels of UFA stimulate de novo milk synthesis and produce milk fat globules that are smaller, have greater surface tension, and are more stable during mechanical processes, such as pumping milk (Wiking et al., 2019). This explains the observation of reduced FFA during the summer months for herds with cows on pasture: fresh pasture feed contains higher levels of UFA (Wiking et al., 2019). Wiking et al. (2019), in 3,585 herds (259 organic), observed that organic herds had a lower concentration of FFA (averaging 0.62 mmol/100 g of fat) than conventional herds (averaging 0.66 mmol/100 g of fat); the differences were most evident during the summer months when cattle were grazing. However, in that study, correlations between feeding regimens and FFA explained only 19% of the variation in FFA concentration (Wiking et al., 2019).

On most dairy farms, milk is harvested using 1 of 3 systems: tie-stalls, parlor, or milking robots. Tie-stalls and parlors are referred to as conventional milking systems, whereas robots are referred to as automated milking systems (**AMS**). An association between milking system and higher levels of FFA has been reported in the literature, with tie-stalls and AMS having, on average, higher FFA levels compared with parlors (de Koning et al., 2003; Wiking et al., 2005, 2006, 2019), although the between-farm variability across all 3 systems is substantial. Wiking et al. (2019) reported that although most (71–100%, depending on the milking system) of their 3,585 study herds had FFA concentrations between 0.8 and 1.3 mmol/100 g of fat, herringbone parlors had the greatest proportion of herds with FFA <0.8 mmol/100 g of fat (12.3% for organic and 14.5% for conventional).

Although tie-stalls are associated with increased FFA concentrations compared with parlor milking systems, a more substantial difference in FFA has been reported between parlors and AMS (de Koning et al., 2003; Rasmussen et al., 2006; Wiking et al., 2006). Rasmussen et al. (2006) reported that milk from 5,614 conventional herds had an average FFA of 0.75 mEq/L, which was lower than that for AMS models (0.77–0.92 mEq/L, depending on the model). de Koning et al. (2003) reported an increase in FFA concentrations from 0.39 to 0.57 mmol/100 g of fat after the 262 conventional parlor farms transitioned to AMS.

Despite milk quality concerns including increased FFA (de Koning et al., 2003; Rasmussen et al., 2006), AMS are becoming more popular in the dairy industry because they can reduce labor, enhance animal welfare, and increase milk yield by up to 18% (Wiking et al., 2019). An increase in milk yield is most likely due to increased milking frequencies because an AMS allows cows to be milked more than twice a day (Wiking et al., 2006, 2019). However, an increased herd average milking frequency in AMS is also the most notable explanation for higher FFA concentrations (de Koning et al., 2003; Wiking et al., 2005, 2006, 2019). Rasmussen et al. (2006) reported a previous study where AMS herds had increased FFA concentration (from 0.42 to 0.71 mmol/100 g of fat) when going from 2 to 3 milkings per day. de Koning et al. (2003) reported that conventional Dutch farms (n = 262) that milked 3 times a day had significantly higher concentrations of FFA than those milking twice a day. Wiking et al. (2006) reported an increase from 1.14 to 1.49 mmol/100 g of fat in milk from 2 teats (half of the udder) that went from being milked twice to 4 times a day. This FFA increase was seen as early as 5 d after the milking frequency increased (Wiking et al., 2006).

Both mechanical and biological theories have been proposed to explain elevated FFA with increased milking frequency (Wiking et al., 2019). One is that the milk fat globule membrane is not given sufficient time to form due to decreased time between milkings (Wiking et al., 2019), which makes it more susceptible to damage and the resultant hydrolysis of TAG to yield FFA. Increased milking frequency is correlated with higher rates of milking failures, and Rasmussen et al. (2006) suggested that this can be another indicator of milk at higher risk for elevated FFA concentration. An increased milking frequency is also associated with a greater proportion of large milk fat globules, which are more unstable and prone to spontaneous lipolysis (Wiking et al., 2006). Wiking et al. (2006) reported that the volume-weighted diameter of the milk fat globules increased from 4.21 to 4.36 μm in 11 cows transitioned from twice to 4 times a day milking.

Individual cow milk yields at each milking are lower with increased milking frequency, which also leads to higher FFA concentrations (Rasmussen et al., 2006). This relationship is especially significant for late-lactation, low-yielding cows (de Koning et al., 2003; Rasmussen et al., 2006). For the 20 cows in their study, Rasmussen et al. (2006) reported an average decrease of 0.16 mmol/100 g of fat in FFA for every additional liter of milk at a single milking.

The opposite association between milk yield and elevated FFA was observed when analyzing total milk yield. Herds with higher-yielding cows are associated with increased levels of FFA (de Koning et al., 2003; Rasmussen et al., 2006). The reason for this is not clear and could be confounded by milking system.

Excess air in the milking system can contribute to elevated FFA concentration (de Koning et al., 2003; Wiking et al., 2005, 2019; Rasmussen et al., 2006). When there is a greater air-to-milk ratio in the milking equipment, the added air can rupture the milk fat globule membrane, creating “bubbling” (de Koning et al., 2003; Wiking et al., 2005; Rasmussen et al., 2006). Ensuring optimal milk yields at individual milkings prevents a high air-to-milk ratio and protects against elevated FFA concentrations (Wiking et al., 2019). Rasmussen et al. (2006) demonstrated that reducing air inlet in the AMS milking cluster of 20 cows from 7 L/min to 1.7 and 0 L/min decreased FFA concentrations from 1.02 to 0.90 and 0.77 mEq/100 g of fat, respectively. Blocking the air inlet completely contributed to higher vacuum fluctuations, but no direct effects on FFA concentration were reported (Rasmussen et al., 2006).

The international standard for total air admission in a cluster is 4 to 12 L/min, but AMS surpass this rate (Rasmussen et al., 2006). In AMS, each quarter milker allows an air admission of 4 to 7 L/min and, because there are 4 quarters per milking unit, the accumulation of air can reach 15 to 28 L/min (Rasmussen et al., 2006). Automatic milking systems likely have an increased air admission requirement due to their individual quarter milkers, long and narrow milk tubes with elevation changes, individual shut-off valves, foremilk separators, and separate milk meters (Rasmussen et al., 2006).

In conventional milking systems, air leakage and excessive air in the cluster are common issues leading to reduced milk quality (Rasmussen et al., 2006). When these are paired with high-line pipelines that require additional air to lift milk, there is an increased risk of air-to-milk contact that may cause mechanical stress on the milk fat globule membranes (Rasmussen et al., 2006; Wiking et al., 2019). Rasmussen et al. (2006) reported that of 31 conventionally milked herds in their study that received a milk inspector visit, 71% had faults in air leakage and 61% had faults related to excess air in the cluster. The conventional farms with air leaks had, on average, FFA concentrations 0.16 mEq/L higher than those of herds with faults other than air leakages (Rasmussen et al., 2006). After inspection, these affected farms' FFA concentrations decreased from 1.82 to 1.32 mEq/L, demonstrating that targeting air admission concerns could reduce FFA concentrations (Rasmussen et al., 2006).

Pumping milk through pipelines to reach the bulk tank induces physical stress on milk fat globules that can result in membrane breakage and release of FFA (de Koning et al., 2003; Wiking et al., 2005, 2019; Rasmussen et al., 2006). The effect on FFA can be amplified when excessive air is present, as in the case of AMS (Wiking et al., 2019). Although there have been no effects of specific pump types and increased FFA reported (Wiking et al., 2019), a longer post-run milk pump has been associated with elevated FFA concentrations (de Koning et al., 2003). This can be a concern in high-line milking systems because of the larger force required to lift and transport milk to the tank (Wiking et al., 2019).

Rasmussen et al. (2006) reported that of 56 study herds visited by a milk inspector, 67% of AMS and 29% of conventional systems were identified as having faults in the pumping of milk. After the milk inspection visit, FFA concentrations were reduced by an average of 0.50 mEq/L of milk, demonstrating the protective effect that proper milk pump function has on reducing FFA concentrations.

Wiking et al. (2005) conducted a study that found an increase in FFA of 1.03 mmol/100 g of fat when milk from 8 mid-lactation Holstein cows was immediately pumped at 31°C compared with 4°C. This can be explained by the coalescence of milk fat globules, which occurs at higher milk temperatures. Lower temperatures induce partial crystallization of the milk fat globule, which stabilizes the milk fat globule membrane and helps it withstand the physical stress during pumping and agitation (Wiking et al., 2019). Wiking et al. (2005) concluded that pumping milk immediately (within 15 min) after cooling to 4°C is important to limit FFA concentrations. Compared with milk that was held for 60 min at 4°C, milk that was pumped within 15 min of cooling to 4°C had lower FFA concentrations, by 0.24 mmol/100 g of fat. This effect can be explained by the adsorption of plasma proteins and thus LPL to the milk fat globules (because the majority of LPL is bound to caseins) during cold milk storage (Wiking et al., 2005, 2019).

Effective precooling mechanisms (if any) implemented on a farm include a plate cooling exchanger to efficiently lower the milk temperature (Wiking et al., 2005, 2019; Rasmussen et al., 2006). Wiking et al. (2019), studying 277 AMS farms, showed a decrease in FFA concentration by 0.25 mmol/100 g of fat for those with a plate cooler compared with those that did not use a plate cooler. Currently in the dairy industry, farms either do not have additional cooling or have a precooling mechanism situated after the milk pump (not before). Therefore, recommendations by Wiking et al. (2005) to install additional cooling could be taken into consideration to potentially decrease FFA concentrations.

Maintaining bulk tank function within regulatory requirements and routine system checks are important to prevent elevated FFA concentrations (de Koning et al., 2003; Rasmussen et al., 2006; Wiking et al., 2019). In AMS, the risk of milk freezing in the bulk tank is more common due to lower milk volumes entering an empty tank and the cooler being turned on too quickly; freezing of milk has been associated with elevated FFA concentrations (de Koning et al., 2003; Rasmussen et al., 2006; Wiking et al., 2019). Rasmussen et al. (2006) reported that 58% of the 24 AMS herds that received a milk quality inspection visit due to FFA concerns had issues with overcooling their milk. After correcting the cooling concern(s), FFA concentration decreased by, on average, 0.52 mEq/L. Similarly, 79% of those AMS herds had issues with excessive bulk tank agitation and, after correction, FFA decreased (Rasmussen et al., 2006). For the 31 conventional systems, resolving agitation concerns resulted in a 0.55 mEq/L decrease in FFA concentrations (Rasmussen et al., 2006). The protective effect in all milking systems emphasizes the importance of correct tank agitation onset and settings to prevent elevated FFA concentrations.

Temperature fluctuations in the bulk tank can add stress to the milk fat globule membrane and risks higher FFA concentrations (de Koning et al., 2003; Wiking et al., 2005, 2019). This can occur in AMS with a buffer tank, where milk may sit for hours during milk pick-up and tank washing (Wiking et al., 2019). When the milk is then transferred (normally by gravity flow) to a hot bulk tank that was recently sanitized, the large temperature difference can increase FFA concentrations (Wiking et al., 2019). In conventional systems, warm milk entering and mixing with cold tank milk can also cause temperature fluctuations that increase FFA concentrations (Wiking et al., 2019). This effect could be minimized with the implementation of a precooling mechanism (Wiking et al., 2005, 2019).

Minimizing and responding to any bulk tank alarms has also been associated with reduced FFA concentration (de Koning et al., 2003). Farms with regular checks to the attention list and cleaning systems were associated with lower FFA concentrations, and those with more cooling alarms were associated with higher concentrations of FFA (de Koning et al., 2003).

The range and number of farm factors contributing to elevated FFA concentrations could explain why the increase in FFA is greatest within the first 24 h of milking (Wiking et al., 2006). It is evident that elevations in FFA concentrations arise from a host of farm factors, including milk production (feeding), milking system, milk transport, and milk storage. Based on this review, we conclude that the major factors associated with increased levels of FFA are non-parlor milking systems, increased air admission, the absence of additional cooling, temperature fluctuations in the bulk tank, and rations high in SFA (de Koning et al., 2003; Wiking et al., 2005, 2006, 2019; Rasmussen et al., 2006). These results can serve as a foundation for future research aimed at maintaining low levels of FFA to ensure milk quality and consumer satisfaction with dairy products.
